# In Search of Oscillatory Traces of the Internal Clock

**DOI:** 10.3389/fpsyg.2016.00224

**Published:** 2016-02-23

**Authors:** Tadeusz W. Kononowicz, Virginie van Wassenhove

**Affiliations:** Cognitive Neuroimaging Unit, CEA DSV/I2BM, INSERM, Université Paris-Sud, Université Paris-Saclay, NeuroSpin CenterParis, France

**Keywords:** time perception, interval timing, internal clock, oscillations, striatal beta frequency

## Interval timing, pacemaker(s), and neural oscillations

Neural oscillations are ubiquitous in the mammalian brain and they are typically classified according to their specific frequency responses (Buzsáki, 2006). Neural oscillations are hypothesized to organize communication within and between brain networks (e.g., Fries, 2015). Neural oscillations have increasingly been associated with various cognitive functions such as attention (Klimesch, 2012), working memory (Gulbinaite et al., 2014a; Haegens et al., 2014), and cognitive control (Cavanagh et al., 2009; Gulbinaite et al., 2014b) but also temporal expectation (Praamstra et al., 2006; Cravo et al., 2011; Rohenkohl and Nobre, 2011) and timing (Treisman, 1963; van Wassenhove, 2009; Kösem et al., 2014; Kononowicz and van Rijn, 2014). One quest in cognitive neuroscience is to explain how neural oscillations can subserve complex cognitive processes. Here, we mainly focus on the role of spontaneous rhythms in interval timing (also see van Wassenhove, in press); however, some hypotheses are supported by the literature on rhythmic entrainment.

**Figure 1:**
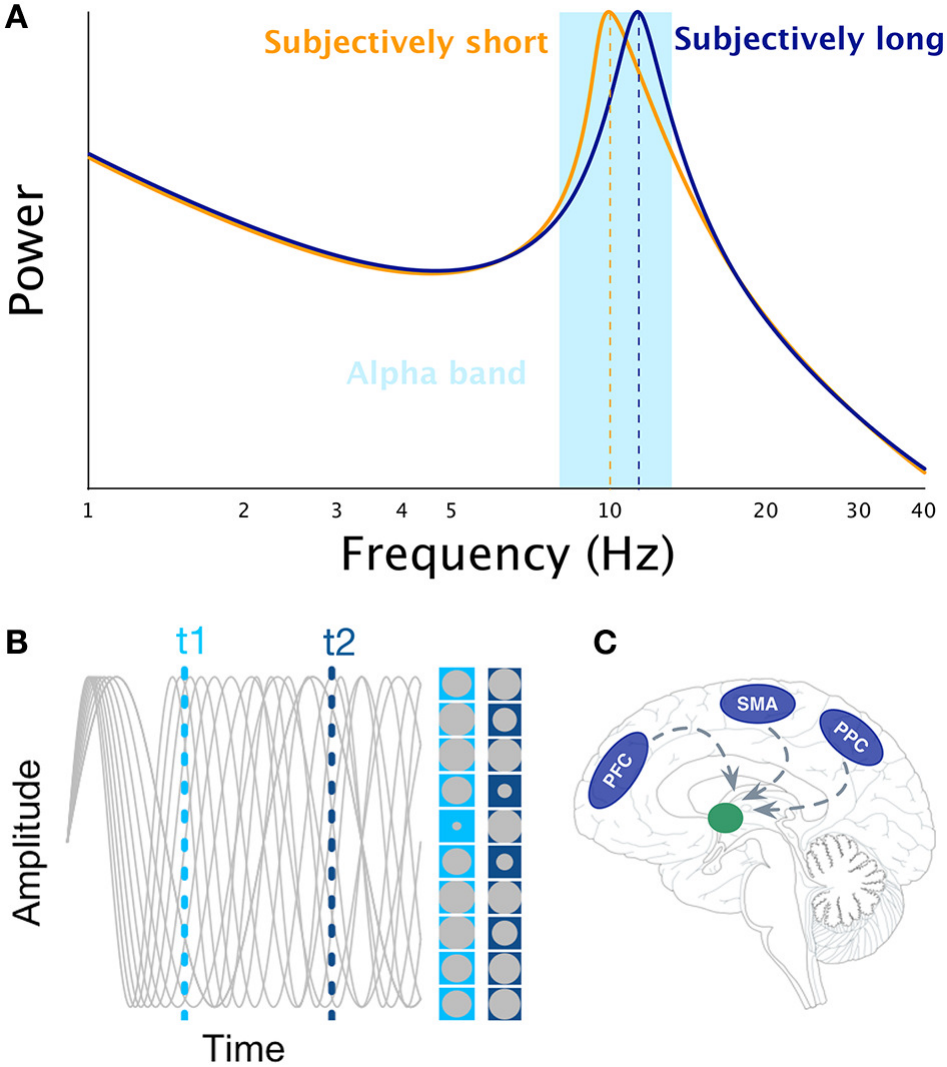
**Illustration of the main interval timing theories of interval timing that rely on the notion of neural oscillations**. Panel **(A)** illustrates the idea that faster alpha rhythms results in longer estimates of time as more pulses could be accumulated in a given physical time interval (Treisman, 1963). Panel **(B)** illustrates the SBF model. The gray sinusoids depict oscillators in an example trial. The amplitude of each oscillator is represented by the size of gray circle at t1 and t2 times, respectively. Panel **(C)** illustrates the main brain regions engaged in interval timing (PFC, SMA, PPC) and their presumed projections to the striatum as suggested by the SBF model.

One of the possible cognitive abilities neural oscillations may support is interval timing (Treisman et al., 1994), which is the ability to perceive, store, encode, and reproduce temporal intervals ranging from few 100 milliseconds to minutes. Over decades, experimental psychologists have proposed the existence of a cognitive mechanism akin to an internal clock. In search for the neural bases of the internal clock(s), it may be tempting to draw an analogy between ticking clocks and oscillating neuronal networks, as one of the reference papers in neurosciences states, “Clocks tick, bridges, and skyscrapers vibrate, neuronal networks oscillate” (Buzsáki and Draguhn, 2004, pp. 1926). Indeed, some interval timing theories have followed through this idea: Treisman (1963) suggested, that the internal clock could consist of a pacemaker which at the beginning of the to-be-timed interval would start sending pulses, that are then stored in the accumulator. The pulse count could serve as a subjective estimate of time. Furthermore, to implement this model into biologically plausible mechanisms, Treisman proposed that the pulse rate of the pacemaker would be driven by neural oscillations in the alpha range (8–12 Hz, see Figure 1A): faster alpha rhythms would thus result in longer estimates of time than slower alpha rhythms considering, that more pulses would accumulate during the same physical time interval (Treisman et al., 1994). As no simple relationships have been found between the rate of visual flicker, neural oscillations, and the subjective perception of duration when using oscillatory entrainment (Herbst et al., 2013, 2014; although see Johnston et al., 2006), it remains plausible that spontaneous fluctuations of alpha peaks could modulate perceived duration. For example, Haegens et al. (2014) have shown, that alpha peak frequency changed as a function of cognitive load in a N-back working memory (WM) task such that the larger the WM load, the higher the alpha peak frequency. These results indicate that subjectively longer durations could be associated with larger alpha peak if WM is implicated in the estimation of duration (Gu et al., 2015; van Wassenhove, in press).

Despite mechanistic attempts to link oscillatory processes with internal clock models, direct implementations of internal clock models still lack solid neural foundations whereas, more biologically grounded frameworks have been more plausible (Buhusi and Meck, 2005).

## The (striatal) beat frequency model

The most prominent neurobiologically plausible model of interval timing is the Striatal Beat Frequency (SBF) model (Matell and Meck, 2000, 2004; Buhusi and Meck, 2005) developed on the basis of the beat frequency model (Miall, 1989). One major assumption of SBF (Figure 1B) is the existence of cortical oscillators of various frequency responses most likely located in the Pre-Frontal Cortex (PFC) which is part of the mesocortical pathway. However, other cortical regions cannot be excluded [e.g., Supplementary Motor Area (SMA), Posterior Parietal Cortex (PPC), or sensory cortices, Figure 1C]. At the onset of an interval to be timed, the model posits, that cortical oscillators are phase-reset and, at the offset of the interval, the state of these cortical oscillators is read out by medium spiny neurons located in the striatum. Hence, the SBF model considers, that the phase of cortical oscillators gives rise to a unique activation pattern over time (Buhusi and Meck, 2005; Oprisan and Buhusi, 2011, 2014) and, that spiny neurons are coincidence detectors reading out the state of these cortical oscillators. Note that in SBF, the pattern of activation is identical whether one reads the phase or the amplitude of the oscillators. Although, cortical oscillators seem to be a key element of the SBF model, only little evidence currently supports the existence of a dedicated set of cortical oscillators for interval timing (e.g., Matell, 2014). It is also unclear whether cortical oscillators are really necessary for the SBF model, as any stable pattern of neural activation (Crowe et al., 2014; Merchant et al., 2014; Mello et al., 2015) within to-be-timed intervals but variable across to-be-timed intervals would be sufficient as input to insure reliable coincidence detection (also see Meck et al., 2013).

## Quantifying the role of cortical oscillators

When considering oscillatory processes in the context of the SBF model, at least two important predictions regarding neural oscillators have to be taken into account. The first prediction is, that in order to provide a meaningful pattern, cortical oscillators have to be phase-reset, such that they always start from the same fixed state. For example, the results by Parker et al. (2014) suggest, that more precise phase reset of ongoing theta oscillations in the medial frontal cortex results in better timing accuracy (Kononowicz, 2015), something that would be in line with the SBF model. This hypothesis awaits future tests and more compelling evidence have to be provided.

The second prediction is linked to the idea, that the speed of internal clock can be modulated by the speed of cortical oscillators (Oprisan and Buhusi, 2014), which are modulated by tonic levels of dopamine (Oprisan and Buhusi, 2011). It is very often assumed, that the clock speed could be represented by the alpha band regime (Treisman et al., 1990, 1994) as it is the most prevalent spontaneous rhythm in the mammalian brain (Oprisan and Buhusi, 2014). However, as previously discussed, the relationship between alpha peak power and fluctuations in subjective timing has not been clearly established; direct attempts to test this hypothesis have not succeeded (Treisman et al., 1990, 1994). The power of alpha is a good marker of temporal expectation (Praamstra et al., 2006; Cravo et al., 2011; Rohenkohl and Nobre, 2011), which is in line with the hypothesized role of alpha as a selective coordinator implicated in the temporal prioritization of sensory events (Jensen et al., 2014). Hence, one possible departure from the early proposals could be that a single dominating frequency may not be necessary to represent the clock speed as neural oscillations outside of the alpha range have been implicated in interval timing (Busch et al., 2004; Kaiser et al., 2007; Sperduti et al., 2011), raising the possibility that other rhythms could serve as “pacemakers.” For instance, recent studies suggest a signifant role of beta oscillations in timing (Iversen et al., 2009; Fujioka et al., 2012, 2015; Bartolo et al., 2014; Teki, 2014; Kononowicz and van Rijn, 2014; Wiener and Kanai, 2016) and the phase characteristics of low-frequency oscillators can predict subjective timing (Cravo et al., 2011; Kösem et al., 2014), suggesting, that different neural oscillations have the potentiality to track time. Therefore, instead of focusing on one single neural oscillation, future studies should explore local trial-to-trial fluctuations across frequency bands and how subdominant frequencies vary as a function of subjectively perceived time intervals. Complementary to this, addressing the implications of such markers at different time scales and across sensory modalities may be desirable.

Interestingly, a recent review by Gu et al. (2015) proposes to unify interval timing and working memory models. Specifically, these authors proposed, that working memory and interval timing can originate from the same oscillatory processes such as gamma and theta oscillations, and phase-amplitude coupling between these frequency bands (Lisman, 2010). The proposed model largely focuses on oscillatory processes that could be shared between working memory and SBF. Nonetheless, the empirical ways to assess the principles of SBF model are still lacking. As the gist of the SBF lies in the notion of communication between cortical areas and the striatum, here we discuss the possibility of testing this hypothesis by investigating functional connectivity between the striatum and PFC.

## Striatum-PFC coupling and the SBF model

Striatal neurons are ideal candidates for coincidence detection as they receive direct inputs from cortical neurons. Through coincidence detection of spiking activity from two or more cortical regions, the same striatal neuron will discharge within a given time window. For instance, Matell et al. (2003) showed, that neural activity in the striatum and the anterior cingulate cortex varied before 10 and 40 s when the reinforcement was presented at one of these two time points, suggesting, that neuronal populations respond to to particular time intervals. However, this pattern although predicted by SBF could largely be confounded by motor activity of lever pressing. Nevertheless, note, that Riehle et al. (1997) observed transient synchronization of neurons in motor cortex when stimuli were expected, but failed to appear. Although, this work is very important it only shows pattern of activity that fits into the SBF model under certain conditions. Given, that an important premise of the SBF model is the communication between striatal neuronal ensembles and cortical neurons, we propose, that investigating functional connectivity between subcortical and cortical structures can serve as an important step extending the results of Matell et al. (2003) and giving further support to the SBF model. For example, Antzoulatos and Miller (2014) found, that perceptual (non-temporal) category learning was accompanied by increased synchronization within beta band range (12–30 Hz) between the PFC and striatum, demonstrating the role of functional connectivity in learning. Specifically, synchronization was larger for correct trials. On the basis of the SBF model, a change of cortical-striatal synaptic weights through learning is predicted to reflect a memory mechanism such as the one implemented in the Scalar Expectancy Theory (Gibbon, 1977). Taken together, striatal neurons are predicted to become more sensitive to firing as a function of specific PFC neurons, and these learning effects should be visible during training of temporal discrimination as a change in inter-areal synchronization. Moreover, according to the SBF model and in line with the results of Antzoulatos and Miller (2014), inter-areal synchronization should be enhanced in “correct” trials (Kononowicz, 2015). Particularly, the striatal-PFC synchrony enhancement should emerge at the time of a standard interval, for example in the task where subjects compare a comparison interval, that could vary in length to a fixed standard interval. That is because striatal and PFC structures should become transiently synchronous due to previous learning enhancing sensitivity/tuning of striatum to the particular neural pattern exhibited at the time of standard interval.

The synchronization of neural oscillations has been associated with neuronal mechanisms such as coincidence detection, neural plasticity though long term potentiation/depression mechanism, and neuronal communication (Fell and Axmacher, 2011). These processes seem like a plausible candidate to coordinate striatum-PFC communication in recognition for specific patterns considered by SBF model. Specifically, the simplest scenario would predict an increase in coherence or spike-filed coherence for accurately timed trials. Coherence was proposed to reflect facilitated communication between brain regions (e.g., Fries, 2015). Effective communication should be linked to the successful timing performance if indeed communication between the striatum and PFC is a key component of timing system as proposed in the SBF model. This cortico-striatal spike-field coherence should be specifically enhanced at the time of standard interval if striatal neurons recognize cortical pattern (see Kononowicz and van Rijn, 2015). Furthermore, the role of cortico-cortical coherence has been shown in passive rhythmical stimulation paradigms, in which an increase in coherence coincided with the next tone occurrence (Fujioka et al., 2012). These results do support the hypothesis sketched in this paper and also suggest cortico-cortical analysis. Moreover, recent progress in neuroscientific methods allows to adress this questions in animals and humans using MEG/EEG modeling (David et al., 2011), but also deep brain recordings.

## Author contributions

All authors listed, have made substantial, direct and intellectual contribution to the work, and approved it for publication.

### Conflict of interest statement

The authors declare that the research was conducted in the absence of any commercial or financial relationships that could be construed as a potential conflict of interest.
